# Transcriptome-wide comparison of sequence variation in divergent ecotypes of kokanee salmon

**DOI:** 10.1186/1471-2164-14-308

**Published:** 2013-05-07

**Authors:** Matthew A Lemay, David J Donnelly, Michael A Russello

**Affiliations:** 1 Department of Biology, University of British Columbia, Okanagan Campus, 3333 University Way, Kelowna BC, V1V 1V7, Canada

**Keywords:** Adaptation, High resolution melt analysis, Next-generation sequencing, Oncorhynchus nerka, Single nucleotide polymorphisms

## Abstract

**Background:**

High throughput next-generation sequencing technology has enabled the collection of genome-wide sequence data and revolutionized single nucleotide polymorphism (SNP) discovery in a broad range of species. When analyzed within a population genomics framework, SNP-based genotypic data may be used to investigate questions of evolutionary, ecological, and conservation significance in natural populations of non-model organisms. Kokanee salmon are recently diverged freshwater populations of sockeye salmon (*Oncorhynchus nerka*) that exhibit reproductive ecotypes (stream-spawning and shore-spawning) in lakes throughout western North America and northeast Asia. Current conservation and management strategies may treat these ecotypes as discrete stocks, however their recent divergence and low levels of gene flow make in-season genetic stock identification a challenge. The development of genome-wide SNP markers is an essential step towards fine-scale stock identification, and may enable a direct investigation of the genetic basis of ecotype divergence.

**Results:**

We used pooled cDNA samples from both ecotypes of kokanee to generate 750 million base pairs of transcriptome sequence data. These raw data were assembled into 11,074 high coverage contigs from which we identified 32,699 novel single nucleotide polymorphisms. A subset of these putative SNPs was validated using high-resolution melt analysis and Sanger resequencing to genotype independent samples of kokanee and anadromous sockeye salmon. We also identified a number of contigs that were composed entirely of reads from a single ecotype, which may indicate regions of differential gene expression between the two reproductive ecotypes. In addition, we found some evidence for greater pathogen load among the kokanee sampled in stream-spawning habitats, suggesting a possible evolutionary advantage to shore-spawning that warrants further study.

**Conclusions:**

This study provides novel genomic resources to support population genetic and genomic studies of both kokanee and anadromous sockeye salmon, and has the potential to produce markers capable of fine-scale stock assessment. While this RNAseq approach was successful at identifying a large number of new SNP loci, we found that the frequency of alleles present in the pooled transcriptome data was not an accurate predictor of population allele frequencies.

## Background

Biology is currently undergoing a genomics revolution [1]. With the increasing ubiquity of next-generation sequencing technology, it is now possible to rapidly generate millions of base pairs of DNA sequence data for a fraction of the per-base cost required for chain-termination Sanger sequencing. The result is an exponential increase in the abundance of genomic resources available for studying non-model organisms [2]. Single nucleotide polymorphisms (SNPs) have emerged as a marker of choice for population-level studies in the genomics era [3-5]. Due to their broad genomic distribution, direct association with functional significance, and ease of genotyping, SNPs represent an improvement over conventional markers such as amplified length polymorphisms (AFLPs) and expressed sequence tag (EST)-linked microsatellites. The efficiency of SNP discovery and validation may be further increased through targeting of the transcriptome (RNAseq; [6]) or restriction-site associated DNA tags (RAD; [7,8]).

The emerging field of population genomics has evolved in tandem with these novel technologies, incorporating genome-wide sequence data into the study of systems that historically would have been limited to a small number of neutral markers [9]. Central to a population genomic approach is the identification of statistical outlier loci that exhibit locus-specific patterns that are highly divergent from the rest of the genome, representing candidate regions under selection [10-12]. The combined signal from high density neutral and putatively adaptive SNPs throughout the genome offers great potential for investigating evolutionary and ecological questions in natural populations. For example, several recent studies have found that the use of outlier loci can reveal fine-scale population structure beyond what was previously inferred from conventional neutral markers (e.g. Atlantic herring [13], Atlantic cod [14], Atlantic salmon [15]). Moreover, population genomic approaches that incorporate statistical outlier loci offer great potential for delineating conservation units [16,17] and informing fisheries management [18].

Kokanee salmon are recently diverged land-locked populations of sockeye salmon (*Oncorhynchus nerka*) that exist in lakes throughout western North America and northeast Asia. Two reproductive ecotypes of kokanee have been described based on spawning behaviour and location. Stream-spawning kokanee migrate into tributaries in early autumn, display pronounced secondary sex characteristics, site defence, and redd formation. Conversely, shore-spawning populations (also known as beach-spawners) breed much later in the autumn, lack secondary sex characteristics and site defence, and mate directly on the shoreline rather than migrating into tributaries [19,20]. In many lakes, the two ecotypes occur in sympatry. Although kokanee ecotypes display different reproductive traits and experience varying incubation environments, outside of the spawning season the ecotypes are panmictic within lakes and morphologically indistinguishable.

Throughout their range, kokanee constitute a heavily managed recreational fishery of great socioeconomic and ecological importance [21]. The ability to distinguish stream- and shore-spawning kokanee is an important goal for fisheries managers, as ecotypes may be differentially impacted by alternative conservation and management regimes with regard to water use, the protection of spawning habitat, and recreational harvest regulations. For example, conservation strategies for kokanee have focused primarily on the preservation of stream-spawning habitat by the formation of spawning channels. However, management efforts for shore-spawning populations are in their infancy and are hindered by a lack of data on the size of shore-spawning populations within lakes. While visual surveys of kokanee during the spawning season are quite accurate in streams, it is considerably more difficult to obtain visual measurement of shore-spawner abundance, especially in lakes where shore populations spawn at greater depth or at night. Kokanee management would be significantly enhanced by the identification of molecular markers capable of assigning individuals to the correct ecotype, however the identification of sufficiently fine-scale markers has been hindered by the recent divergence and correspondingly weak genetic structure of the two ecotypes [18].

Previous studies using neutral loci have found evidence for low levels of genetic differentiation based on mitochondrial DNA haplotype and microsatellite loci, yet individual assignment probabilities to ecotype were generally low [22-24]. More recently, work using a panel of EST-linked microsatellite loci has identified a set of markers with a high association of genetic diversity with reproductive ecotype, suggesting that there may indeed be a genetic basis for the evolution of kokanee ecotypes [18]. However these markers suffer from poor coverage across the genome and provide little information on the specific genes involved in the divergence of the two ecotypes.

Here, we evaluated an RNAseq approach for preferentially identifying SNPs of putatively adaptive significance within and among kokanee ecotypes. We used the Roche 454 GS FLX Titanium platform to generate transcriptome-wide sequence data for pooled cDNA libraries of shore- and stream-spawning kokanee. These data were used for SNP discovery and subsequent validation in natural populations, enabling explicit investigations of ecotype-specific patterns of genetic variation.

## Results and discussion

### Sequencing and assembly

**Figure 1:**
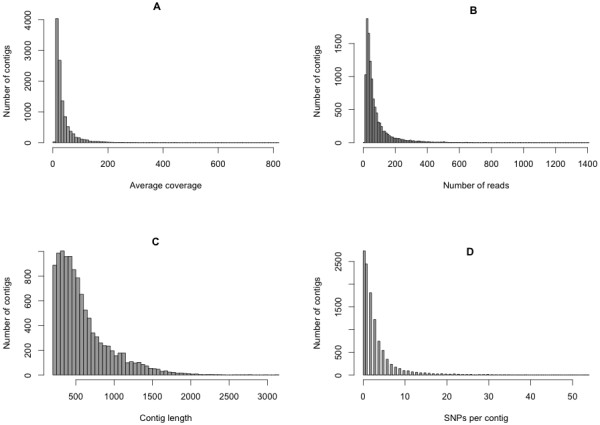
**Characterization of the contigs present in the high coverage data set.** Histograms represent (**A**) average coverage of each contig (mean = 37.0), (**B**) number of reads (mean = 77.9), (**C**) contig lengths (mean = 594.8 bases), and (**D**) the number of SNPs for each of the high coverage contigs.

We generated ~750 million base pairs of sequence data corresponding to 1.3 × 10^6^ and 1.4 × 10^6^ reads for the shore and stream-spawning cDNA libraries, respectively, with an average length of 271 bases per read (Table 1). A *de novo* assembly was carried out with the trimmed reads from both ecotype libraries in order to generate contigs to use as reference sequences; this assembly incorporated 87% of the transcriptome reads to produce 123,547 contigs (Table 2). We then mapped the raw reads back to these reference contigs separately for each ecotype and generated a refined data set consisting only of contigs that had a minimum average coverage of 5× for each ecotype and a minimum length of 200 bases. The resulting data set (hereafter referred to as the high coverage data set) was retained for downstream analyses (Table 2; Figure 1; Additional file 1 and Additional file 2).

**Table 1:** Summary of next-generation sequence data obtained for each ecotype of Okanagan Lake kokanee

	**Shore-spawner library**	**Stream-spawner library**
No. of bases	371,876,524	373,169,057
No. of reads	1,343,483	1,406,375
Mean read length	276.8 bases	265.3 bases

**Table 2:** Summary of the contigs present in each kokanee data set

	**Total contigs**	**High coverage data set**^**1**^	**Shore-unique contigs**^**2**^	**Stream-unique contigs**^**2**^
No. of contigs	123,547	11,074	277	557
Mean coverage	7.5	37.0	6.8	8.4
Mean length	463.7	594.8	374.2	404.2
Mean no. of reads	14.2	77.9	8.5	12.7

^1^ In the high coverage data set each contig has a minimum length of 200 bases and a minimum of 5× coverage for each ecotype. In addition, this data set has duplicate contigs and contigs that map to pathogen DNA sequences removed.

^2^ Contigs composed of reads from a single ecotype. Minimum length = 200 bases; minimum coverage = 5×. Contigs that map to pathogen DNA sequences have been removed.

### Transcriptome analyses and annotation

A genome duplication event in the early evolution of salmonids and subsequent diploidization has resulted in a genome in which isoloci and paralogous gene copies are common [25]. The presence of paralogous sequence variants (PSVs) among contigs would impede our ability to identify sequence variants between the two ecotypes. To address this problem, we used conservatively high similarity parameters during the *de novo* assembly in order to minimize the incorporation of PSVs within each contig [26,27]. In doing so, two separate but highly similar contigs may be created: one corresponding to functional coding sequence, the other to a PSV. To test for these redundancies in the data, a *de novo* assembly with a slightly reduced sequence-similarity value (0.90) was carried out on the high coverage contigs in order to identify any contig sequences that either partially or totally overlapped. Of the 13,593 contigs initially retained in the high coverage data set, 1,864 (13.7%) aligned with one other contig, and 643 (4.7%) aligned with two or more other contigs. These contigs were discarded from the high coverage data set. The remaining 11,085 contigs (81.5%) were unique and did not show similarity with any other contig.

The strict similarity value used to create the reference contigs may also explain the relatively short size of the contigs retained in the high coverage data set (average length = 595 bases). While the short contig size may limit some down-stream analyses, the minimization of PSVs in the data was of higher priority when optimizing the assembly parameters.

**Figure 2:**
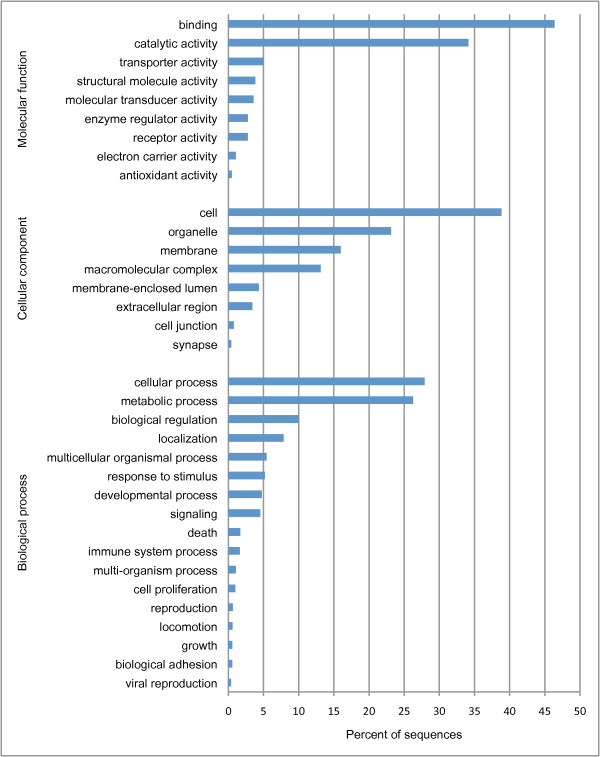
**Functional annotation of the high coverage contigs.** The frequency (%) of each observed gene ontology (GO) term is given for the three GO domains (biological process, cellular component, and molecular function).

A BLAST search of all contigs in the high coverage data set (n = 11,085) produced 4,410 positive matches to sequences in the NCBI database (minimum e-value cut-off = 10^-6^; average e-value = 1.9×10^-9^). Of those contigs with positive BLAST hits, 60% matched published salmonid sequences (contigs matching *Oncorhynchus sp.* = 628; *Salmo sp.* = 1993; all other salmonids = 12). In addition, eleven contigs were a positive match to the infectious hematopoietic necrosis virus (IHNV), a lethal virus that is enzootic in western North America and can have detrimental impacts on salmonid aquaculture [28]. These 11 contigs were subsequently removed from the high coverage data set for all further analyses. Of the high coverage contigs that had positive matches from the Blastx search, 2,285 were subsequently annotated with one or more gene ontology (GO) terms (Figure 2, Additional file 3).

### Mitochondrial genome

There was coverage across all genes in the sockeye salmon mitochondrial genome [GenBank: EF055889], representing 0.3% of the trimmed transcriptome reads (n = 6,824).

### SNP detection

Within the high coverage data set, 8,339 contigs contained SNPs that fell within our detection parameters (minimum coverage 8×, minimum variant frequency 10%, minimum reads per allele = 2, minimum central quality 20). From these contigs, we identified 32,699 putative SNPs that may be used for population genetic analyses of kokanee (Additional file 4). Although there has been much focus on marker development in anadromous sockeye salmon [29-32], to our knowledge, this is the first study that has identified SNPs specifically for reproductive ecotypes of kokanee.

Given that low levels of molecular divergence among ecotypes of Okanagan Lake kokanee have been observed in previous studies, we expected the majority of loci to display similar allele frequencies in both ecotypes. Of the putative SNPs identified in this study, 7,931 were polymorphic in both ecotypes, 12,835 were polymorphic in the stream ecotype but fixed in shore, and 11,933 were polymorphic in the shore ecotype but fixed in stream. There were no SNPs within our detection parameters that were fixed for alternate alleles in the two ecotypes.

In this study, the high frequency of loci that appear to be fixed in one ecotype may be artificially inflated as a result of the small sample size (only eight individuals per ecotype) in the pooled transcriptome libraries and/or due to the SNP detection parameters, which required a minimum of eight reads at a given site and at least two reads for each allele. If one ecotype had coverage below these cut-off values, then it would erroneously appear to be fixed at that site, even if there was some variation present. This could result if one ecotype under-expressed a given gene, preventing it from being detected at sufficiently high levels in the transcriptome data. These potential biases reflect the trade-off between avoiding false SNPs resulting from sequence error, while attempting to account for all possible variation in the data.

The difference in the frequency of the major allele between ecotypes ranged from <1-88% (mean = 19%). Ninety-five percent of all SNPs were contained within a divergence value of 44% or less, suggesting that the overall level of nucleotide divergence is low. The remaining 5% of loci (n = 1,493) represented the most divergent SNPs, with values between 45–88%. These highly divergent SNPs may represent promising targets for ecotype discrimination, potentially associated with genes underlying ecological diversification.

### SNP validation

Primer pairs were designed for 36 loci such that they amplified a 60–200 base pair fragment containing a single SNP (Additional file 5). Of these loci, 18 exhibited successful PCR amplification, were free of introns, and produced sufficiently clear high resolution melt (HRM) signal to attempt the subsequent genotyping validation. These loci were then used to genotype 32 stream-spawners, 32 shore-spawners, and 24 anadromous sockeye salmon using HRM analysis. From the panel of 18 SNPs for which broad-scale HRM genotyping and Sanger validation was attempted, nine loci produced consistently scorable HRM clusters and were retained for downstream analyses.

Sanger sequence data for the remaining loci confirmed that the expected SNP site was indeed polymorphic, however the resulting HRM clusters were not sufficiently discrete to enable accurate genotype assignment. For these loci, Sanger sequencing identified either multiple melt curves with the same genotype or multiple genotypes within the same melt curve. Some of the inconsistencies with the HRM data may be explained by the reduced accuracy of HRM compared with other conventional genotyping methods such as TaqMan® assays [33]. Martino et al. [33] found that rare genotypes and low minor allele frequencies decreased the accuracy of HRM analysis. Both of these factors are likely present in our data given that we preferentially chose divergent loci that were fixed for a single allele in one population. The presence of additional polymorphic sites within the amplicon [26] and the use of loci containing Class 3 (C/G) or Class 4 (A/T) SNPs [34] may also have been factors resulting in weakly differentiated clusters. Given that Sanger sequencing confirmed the presence of the expected polymorphism, we conclude that the failed assays were not due to errors with the initial SNP detection but rather reflect the limitations of the HRM assays at those loci.

There was no evidence of linkage disequilibrium among any of the nine loci for which genotypic data were obtained. One locus (*One74958*) showed a significant deviation from Hardy-Weinberg equilibrium (HWE) in all three populations tested (stream-spawning kokanee, shore-spawning kokanee and anadromous sockeye salmon). This may indicate the presence of a PSV at this locus [26]. One additional locus showed a significant deviation from HWE among the shore-spawning samples only (Table 3). Among the anadromous sockeye salmon samples used in this study, all SNPs showed similar patterns of allele frequencies to kokanee. These results indicate that SNP loci developed from the kokanee transcriptome data could also be useful for genotyping populations of anadromous sockeye salmon.

**Table 3:** Genetic diversity estimates from loci that were successfully genotyped using High Resolution Melt Analysis (HRMA)

**Locus**	**H**_**E**_**/H**_**O**_			**Major allele**^**1 **^**frequency from HRMA (frequency predicted from transcriptome data)**		
	** Stream**	** Shore**	** Sockeye**	**Stream**	**Shore**	**Sockeye**
*One74958*	0.13 / 0.00 *	0.32 / 0.00 *	0.19 / 0.05 *	0.93 (1.00)	0.80 (0.48)	0.90
*One81166*	0.29 / 0.29	0.32 / 0.40	0.41 / 0.48	0.18 (1.00)	0.20 (0.46)	0.28
*One81284+*	0.47 / 0.57	0.43 / 0.61	0.47 / 0.57	0.74 (1.00)	0.70 (0.54)	0.63
*One81385*	0.20 / 0.22	0.38 / 0.36	0.19 / 0.22	0.79 (0.52)	0.75 (1.00)	0.90
*One113434*	0.32 / 0.40	0.32 / 0.40	0.00 / 0.00	0.80 (1.00)	0.80 (0.77)	1.00
*One73115*	0.43 / 0.52	0.44 / 0.47	0.16 / 0.17	0.78 (0.42)	0.67 (0.79)	0.91
*One74836*	0.49 / 0.37	0.50 / 0.53	0.43 / 0.46	0.58 (0.33)	0.58 (0.65)	0.32
*One24190*	0.22 / 0.25	0.35 / 0.45	0.44 / 0.48	0.88 (0.31)	0.77 (0.85)	0.67
*One73476*	0.50 / 0.61	0.50 / 0.25 *	0.50 / 0.57	0.57 (0.35)	0.56 (0.70)	0.52

*Denotes significant deviation from HWE following sequential Bonferroni correction.

^1^ The identity of the major allele is defined as the allele with the highest frequency in the transcriptome data.

+The contig from which this locus was created had some overlap with one other contig (34452). Both contigs were subsequently removed from the high coverage data set. As the overlap did not impact the SNP site, this locus has been retained.

The frequency data obtained from HRM genotyping suggests that while the transcriptome data set provides a valuable tool for identifying novel SNPs, it is limited in its ability to infer population allele frequencies (Table 3). Small sample size in the pooled transcriptome libraries likely contributed to the observed inconsistencies between HRM genotypes and the allele frequencies predicted from the transcriptome data. We initially hypothesized that the most divergent SNPs in the transcriptome sample could indicate high-confidence targets for genetically discriminating ecotypes. Based on these results, however, we feel that the relative frequency of alleles present in the pooled transcriptome reads is not an appropriate surrogate for large-scale SNP genotyping followed by statistical outlier tests. The use of greater sample sizes within the pooled cDNA libraries may improve this outcome. Likewise, emerging protocols that utilize combinatorial labelling methods and RAD tags may provide more efficient and cost-effective alternatives for simultaneously discovering SNPs in non-model organisms and genotyping population-level samplings [7,8].

### Ecotype-unique data

We also generated additional data sets with contigs that contained only reads from a single ecotype (Table 2, Additional file 6). The presence/absence of ecotype-specific contigs may represent candidates for differences in gene expression (rather than sequence variation) between ecotypes. Interestingly, we observed twice as many ecotype-unique contigs from the stream-spawning DNA library, which may indicate a number of genes that are not expressed by the shore ecotype during spawning. However, this discrepancy in ecotype-specific contigs could partially be accounted for by genes with very low levels of expression that were observed in one library by chance alone. A study specifically designed to examine differences in levels of gene expression may be useful at identifying divergence between the two ecotypes.

BLAST searches (Blastn, NCBI, max e-value = 10^-05^) of the ecotype-unique contigs produced 150 positive matches in the shore-unique data set (mean e-value = 1.1×10^-07^) and 393 positive hits for contigs that are unique to the stream ecotype (mean e-value = 2.1×10^-06^). Interestingly, 12 stream-unique contigs (2%, including the highest coverage contig) were a match to *Saprolegnia ferax* [GenBank: AY534144], a pathogenic water mould that is associated with pre-spawning mortality in salmonids [35]. Contigs matching this pathogen were absent among the shore-unique contigs. Similarly, 92 stream-unique contigs (14%) were a match to *Flavobacterium psychrophilum* [GenBank: AM398681], which is a bacterial infection associated with high levels of salmonid mortality [36]. Again, there were no matches to this pathogen among the shore-unique contigs. A similar pattern was also observed for contigs matching *Gyrodactylus salmonis* [GenBank: JN230351], a pathogenic flatworm [37].

To test the possibility that there were reads matching these pathogens that had not been assembled into contigs, we mapped the raw transcriptome reads from each library to the NCBI reference sequence from each of these pathogens. This assembly supported the prevalence of pathogen sequences among the stream ecotype transcriptome data (Table 4). The exception was IHNV, which had been identified in the high coverage data set and was expected to be present in both ecotypes.

**Table 4:** Number of next-generation sequencing reads that aligned to reference sequences from four salmonid pathogens

**Pathogen**	**Number of reads aligned**	
**[GenBank accession]**	**Stream-spawner library**	**Shore-spawner library**
*Flavobacterium psychrophilum*	7,393	219
Complete genome		
[AM398681]		
*Gyrodactylus salmonis*	17	0
Partial ITS1, complete 5.8S rRNA gene, partial ITS2		
[JN230351]		
*Saprolegnia ferax*	393	1
Mitochondrion, complete genome		
[AY534144]		
*Infectious hematopoietic necrosis virus*^1^	285	327
Glycoprotein (G) and non-virion protein (NV) genes		
[IHNGNVJ]		

^1^ IHNV was identified in the high coverage data set containing reads from both ecotypes and was not expected to show ecotype specificity.

Each of these pathogens is associated with reduced fitness in salmonids, not only by killing adults before they are able to spawn [35], but also by persisting in the spawning location and infecting emerging juveniles in the next season [36]. A reduction in pathogen sequences present among kokanee collected in shore-spawning habitats suggests the possibility that shore-spawning behaviour may have evolved, in part, as a way to reduce the pathogen load. While highly speculative, this hypothesis is consistent with other studies (Frazer and Russello, in review), which detected outlier loci associated with immune response in kokanee. Further, the fact that only internal organs and subcutaneous muscle tissue were sampled increases the probability that the observed pathogen sequences were indeed present within the fish tissue, rather then being environmental contaminants that could occur if external fin-clips or operculum punches had been collected. While the present study was not explicitly designed to address this question and may be influenced by stochastic factors, our preliminary results warrant future research to quantitatively compare the pathogen load among kokanee within each of the two spawning habitats.

**Figure 3:**
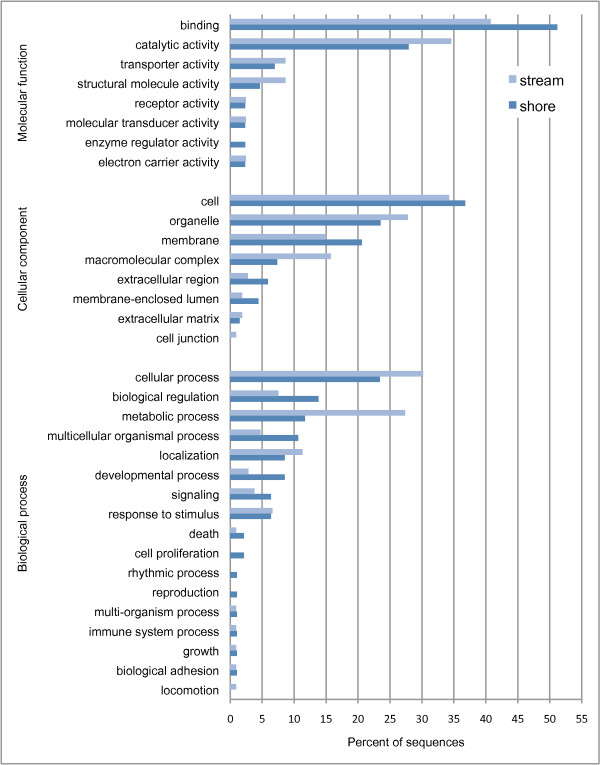
**Functional annotation of contigs that were unique to each ecotype.** The frequency (%) of each observed gene ontology (GO) term is presented for both ecotypes.

All contigs that matched pathogen sequences where removed from the ecotype-unique data sets prior to down-stream analyses. Blast2Go was then used to assign GO annotations to Blastx matches (e-value threshold = 10^-6^) among the remaining contigs that were unique to each ecotype. This analysis found sequence similarity matches for 160 and 76 contigs from the stream and shore data sets, respectively. From these contigs with positive BLAST matches, 64 stream contigs and 34 shore contigs were subsequently annotated with at least one GO term (Figure 3).

## Conclusions

In this study, we harnessed next-generation sequencing technology in order to compare transcriptome-wide patterns of sequence variation among divergent ecotypes of kokanee salmon. We identified 32,699 putative SNPs that could be used for population genetic and genomic studies of both kokanee and anadromous sockeye salmon. We further detected contigs that were unique to each ecotype, which may be indicative of differential gene expression, as well as preliminary evidence for variable pathogen load associated with spawning location.

## Methods

### Sample collection, RNA extraction, and next-generation sequencing

Kokanee were collected during spawning from four stream-spawning sites and three shore-spawning sites in Okanagan Lake, British Columbia (Additional file 7). Animal collections and tissue sampling complied with University of British Columbia animal care protocol #A11-0127 and British Columbia Ministry of Environment collection permit #PE10-66394. A total of eight individuals per ecotype were immediately sacrificed upon collection and dissected in the field. For each of these individuals, five different tissues (heart, liver, muscle, gonad, and olfactory bulb) were harvested and immersed in separate 5ml screw-cap vials containing 2.5 ml of RNALater® (Life Technologies) solution. Samples were held at 4°C for 24 hrs and then stored at −20°C until needed. RNA was extracted from each tissue type using the RNEasy Universal MiniKit (Qiagen) following the manufacturer’s protocol.

Two normalized cDNA libraries (Evrogen, Russia) were constructed using pooled RNA from all shore-spawners (5 tissues × 8 individuals) and all stream-spawners (5 tissues × 8 individuals). The two resulting cDNA libraries were each subject to a full run of 454 GS FLX Titanium sequencing at the Genome Quebec core facility. Pooling of multiple individuals in each sample was used to provide a preliminary indication of genetic variation within and among ecotypes; the combination of five tissue types for each individual was used to maximize the diversity of expressed genes present in each library. RNA samples were normalized to increase the likelihood that rare transcripts were detected in the sequence data. The raw read data from each library was deposited at the NCBI Sequence Read Archive (project # SRP021088) with the following accessions: Stream reads SRR827512, SRR827513; shore reads SRR827572, SRR827573.

### Assembly

The CLC Genomics Workbench (CLC Bio) v.4.8 was used for initial read trimming. Low quality (quality limit 0.05) and very short reads (<100 bases), terminal nucleotides (five from each end), and 454 sequencing adapters and primers were removed from the data set prior to assembly. A *de novo* assembly was then carried out using CLC Genomics Workbench v.4.8 (similarity = 0.96, length fraction 0.5) in order to generate reference contigs for subsequent analyses. A conservatively high similarity value was used in our assembly in order to minimize the incorporation of PSVs within these reference contigs [26,27].

To facilitate a comparison of sequence variation between the two ecotypes, the consensus sequence from each contig created during the *de novo* assembly was used as a reference to map the stream and shore reads separately (CLC Genomics Workbench v.5.5; similarity = 0.96, length fraction 0.5). We retained only those contigs with a minimum length of 200 bases and an average coverage greater than 5× for each ecotype (hereafter referred to as the high coverage data set).

We also generated data sets containing those contigs that were composed of reads from only a single ecotype (same parameters as above, minimum coverage = 5×; minimum length = 200 bases). These two ‘ecotype-unique’ data sets may suggest target genes for subsequent studies examining differences in gene expression among ecotypes.

### Mitochondrial genome

While there is not yet a fully assembled and annotated salmon nuclear genome, an annotated mitochondrial genome sequence is available for most salmonid species. To assess the prevalence of mitochondrial genes in the transcriptome data, we used the mitochondrial genome for sockeye salmon [GenBank: EF055889] as a reference to map the kokanee transcriptome reads (CLC Genomics Workbench v.4.8, similarity = 0.90).

### Transcriptome analysis and annotation

The high coverage data set was subject to sequence similarity searches using Blast2Go v.2 [38,39]. For this analysis, a Blastx search was performed using the NCBI nr database (e-value threshold = 10^-6^, HSP length cut-off = 33). The top five hits for each contig were retained. Following the Blastx search, GO analysis was carried out in order to obtain hierarchical structure information with respect to the three GO domains (molecular function, biological process, and cellular component). This analysis was carried out using Blast2Go v. 2 (e-value threshold = 10^-6^, HSP length cut-off = 20, GO weight = 5).

Sequence similarity searches were also carried out for all contigs in each of the ecotype unique data sets. Both Blastx (same parameters as above) and Blastn (maximum e-value threshold = 10^-5^) searches were performed. Blast2Go v.2 was again used to assign GO annotations to contigs (same parameters as above).

A *de novo* assembly (CLC Genomics Workbench v5.5; similarity = 0.90, length fraction = 0.5) was carried out using all contigs in the high coverage data set in order to identify contigs with overlapping portions. Redundancies among the contigs may indicate the presence of PSVs or be indicative of alternative splicing within the transcriptome data. Contigs that overlapped with one or more other contigs were removed from the high coverage data set.

### SNP discovery

The working data set of high coverage contigs was screened for SNPs using the CLC Genomics Workbench v. 5.5 (minimum coverage 8×, minimum variant frequency 10%, minimum reads per allele = 2, minimum central quality 20). By carrying out mapping and SNP detection separately for the two ecotypes and then combining the resulting SNP tables, it was possible to determine the number and percent of reads that matched the reference contig sequence at each SNP site. The absence of a polyorphism (fixation) in one ecotype was evident as either 100% of reads matching the reference (in this case no SNP would be observed in the table for this ecotype) or 100% of reads possessing a nucleotide other than that of the reference sequence. For example, a fixed difference for a given location would be scored when one ecotype was 100% different from the reference contig sequence, while the other ecotype displayed no polymorphism at that site (i.e. 100% match to the reference). Using this approach we were able to characterize all SNPs as being either: (a) polymorphic in both ecotypes; (b) fixed in one ecotype, polymorphic in the other; or (c) fixed for different alleles in each ecotype. Polymorphic sites with more than two alleles and sites with read ambiguity were removed from the data. Variation in the form of indels was not examined in this study.

A divergence value (based on the index implemented in Juekens et al. [40]) was calculated for each SNP, defined as the absolute value of the difference in the frequency of the major allele among ecotypes. For example, a SNP that was fixed for a different allele in each ecotype would have a divergence value = 1.0; a SNP where the major-allele was fixed in shore and had a frequency of 60% in stream would have a divergence value = 0.40. The approach was used to putatively identify the most divergent polymorphic sites within the transcriptome.

### SNP validation

A subset of 36 SNPs was used to genotype an independent sample of kokanee in order to validate this ascertainment procedure. Validation of candidate SNPs was carried out following a pipeline similar to that implemented by Seeb et al. [26]. Briefly, primers were designed using Primer3[41] such that they would amplify a ~60-200 bp fragment that encompassed a single SNP. Loci that produced a single clean amplicon with no detectable introns were used to genotype 88 individuals using HRM analysis (see below).

Each PCR reaction contained 1.25 μl of 10× buffer, 1.25 μl of 2 mM dNTP mix, 0.5 μl of 10 mM forward and reverse primer, 0.5 units of Kapa *Taq* polymerase (Kapa Biosystems), 20–100 ng of DNA template, and ultra pure water for a total reaction volume of 12.5 μl. For each reaction, a touchdown PCR procedure was implemented using a Veriti thermal cycler (Applied Biosystems). The program had an initial denaturation at 94°C for 2 minutes, followed by 10 cycles at 94°C for 30 seconds, 60°C for 30 seconds, and 72°C for 30 seconds, with the annealing temperature decreasing by 1.0°C per cycle. This was followed by 25 cycles at 94°C for 30 seconds, 50°C for 30 seconds, and 72°C for 30 seconds. The final cycle had an extension of 72°C for 2 minutes and was then held at 4°C. PCR products were run on a 1.5% agarose-gel in order to obtain a preliminary assessment of the quality and size of the amplicon. Loci that showed evidence for the presence of introns (larger PCR products then expected), or had multiple bands were not retained for subsequent analyses.

Loci that produced a single clean PCR product of the anticipated size were then evaluated by HRM analysis using DNA samples from 32 stream-spawners and 32 shore-spawners from Okanagan Lake. For detailed sampling methods for the kokanee DNA samples see Russello et al. [18]. In addition, 24 anadromous sockeye salmon DNA samples were included in this analysis. These samples were collected by the Okanagan Nation Alliance from Osoyoos Lake, British Columbia, which is the closest extant population of sockeye salmon from the same drainage system as Okanagan Lake. Fin clips were removed from mature adults during the spawning season in September 2011. DNA was extracted using a NucleoSpin Tissue Kit (Macherey Nagel) following the manufacturers suggested protocol for 96 well plates.

Each HRM reaction contained 7.2 μl of Precision Melt Supermix (Bio-Rad), 0.4 μl of each primer, 20–100 ng of DNA template, and ultra pure water for a total reaction volume of 20 μl. HRM analyses were run in 96 well plates on a Bio-Rad CFX96 Touch™ real time PCR detection system. A two-step touchdown PCR protocol was used starting with an initial denaturation step at 95°C for 2 minutes, followed by 9 cycles of 95°C for 10 seconds, 60°C for 30 seconds, with the annealing temperature decreasing by 1°C per cycle. This was followed by 43 cycles of 95°C for 10 seconds and 50°C for 30 seconds. The final PCR cycle consisted of 95°C for 30 seconds followed by 55°C for 1 minute. A plate read was obtained at the end of every PCR cycle. The melt curve data were obtained starting at 70°C and increasing by 0.2°C every 10 seconds to a maximum of 95°C. During the melting stage a plate read was obtained at every 0.2°C increment. Melt curve data were analyzed using the Bio-Rad CFX proprietary software.

SNPs that demonstrated multiple clusters of HRM curves (i.e. polymorphic loci) were subjected to Sanger re-sequencing on an ABI 3130 Genetic Analyzer to confirm their genotype. Given that each HRM cluster should represent a single SNP genotype, 1–2 individuals from each cluster were sequenced in order to determine the genotype of each cluster.

For each locus that was successfully genotyped, we calculated expected and observed heterozygosity, and tested for deviations from Hardy-Weinberg and linkage equilibrium. These analyses were carried out using GenePop v.4 [42] followed by a sequential Bonferroni correction for multiple tests [43]. In addition, the allele frequencies observed from HRM genotyping of each locus were compared with the frequency expected from the pooled transcriptome read data. For consistency, the major-allele was defined as the allele with the highest frequency observed in the transcriptome data.

## Competing interests

The authors declare that they have no competing interests.

## Authors’ contributions

MR and ML designed the study. ML collected tissue samples, carried out lab work, analyzed data and drafted the manuscript. DD carried out lab work and assisted with data analysis. MR obtained the funding, assisted with analyses, and helped draft the manuscript. All authors read and approved the final manuscript.

## Supplementary Material

Additional file 1 **High coverage contig sequences.** In FASTA format containing the sequence of all high coverage contigs used for SNP detection (minimum length = 200 bases, minimum coverage = 5× for each ecotype). Click here for file

Additional file 2 **Characterization of high coverage contigs.** Listing the length, coverage, number of reads, and top Blastx hit (NCBI) for each of the high coverage contigs. Click here for file

Additional file 3 **GO terms for each high coverage contig.** Listing the contig name and GO annotation information for each of the three GO domains: Biological process (BP), molecular function (MF), cellular component (CC). Click here for file

Additional file 4 **SNP information.** Characterizing the 32,699 SNPs identified in the high coverage data set. Click here for file

Additional file 5 **SNP primers.** Containing the primer sequences and locus information for all loci for which HRM validation was attempted. Click here for file

Additional file 6 **Ecotype unique contig sequences.** In FASTA format containing the sequence of all contigs that were composed entirely of reads from a single ecotype (minimum length = 200 bases, minimum coverage = 5×). Click here for file

Additional file 7 **Sample collection.** Containing the sampling locations of all kokanee used to generate the two pooled transcriptome libraries. Click here for file
