# Supplementary material for: Transcriptome-wide comparison of sequence variation in divergent ecotypes of kokanee salmon
Source: BMC Genomics. 2013 May 7;14:308. doi: 10.1186/1471-2164-14-308 (PMC3653777; doi:10.1186/1471-2164-14-308)
Supplement: Additional file 5 — SNP primers. Containing the primer sequences and locus information for all loci for which HRM validation was attempted. [file 1471-2164-14-308-S5.doc]

| Locus Name | Contig | Position | Primers | Amplicon size | Alleles | NCBI Accession  (e-vlaue) | Gene |
| --- | --- | --- | --- | --- | --- | --- | --- |
| *One34452* | 34452 | 166 | F: GGCTGTATCTACCCCAAGCA | 143 | A/C | No matches |  |
|  |  |  | R: GTGGGTGCCTTCCTTCTGT |  |  |  |
| *One116118* | 116118 | 148 | F: AGCCTGGAACCAACAAACAC | 133 | C/G | BT075674  (1.28E-27) | Microsomal glutathione S-transferase 1 putative mRNA |
|  |  |  | R: GTGTGAGAAGATGCCACCAG |  |  |  |
| *One27532* | 27532 | 769 | F: TACTGGATTGTACGGATCATCA | 171 | A/G | BT075044  (4.48E-140) | Phosphoglycerate mutase 1 putative mRNA |
|  |  |  | R: TGATGGTCCTGATTCTGTGTTT |  |  |  |
| *One46926* | 46926 | 590 | F: CATACCGCACCAATGGTAGA | 143 | G/T | BT073488  (0) | High mobility group protein |
|  |  |  | R: CATAGCATTAAAACAAGTATGTAGGC |  |  |  |
| *One81284* | 81284 | 168 | F: GCTAGTGAGACAAACATATCACAGTC | 145 | A/T | FJ356141  (4.92E-10) | Conserved noncoding element |
|  |  |  | R: TGGGGCAGAGATATTGGAAC |  |  |  |
| *One74958* | 74958 | 1198 | F: ATGAGGGACTGTGTCGGTTC | 136 | G/T | BT045835  (0) | Cell division control protein |
|  |  |  | R: GACGCATGTGCCAGCTTAC |  |  |  |
| *One81385* | 81385 | 197 | F: GCCATATGGAATAAATCCAGAAA | 126 | G/T | No match |  |
|  |  |  | R: CTGGGACATCATGACCACTTT |  |  |  |
| *One81102* | 81102 | 134 | F: TTGAAAGTGCTCTGGGATTTC | 108 | A/T | BT059875  (2.15E-85) | Fatty acid-binding protein |
|  |  |  | R: CACAGGTGTGGGTTTTGCTA |  |  |  |
| *One72373* | 72373 | 239 | F: TGACGAGACATCATCCGAGA | 141 | C/T | EF055889  (0) | Mitochondrion, complete genome |
|  |  |  | R: AGCGTGGTAGAAGGCTCAGA |  |  |  |
| *One31106* | 31106 | 91 | F: CAAGAATAACACCACAGCTTGA | 121 | A/T | No match |  |
|  |  |  | R: TGAAATCCCAAATGTATGTGTGT |  |  |  |
| *One82069* | 82069 | 568 | F: TGCTTCCTCAGGCTAGTCGT | 141 | G/T | EF055889  (0) | Mitochondrion, complete genome |
|  |  |  | R: CCACCTCATTCACTGCCATT |  |  |  |
| *One82280* | 82280 | 499 | F: CCACATAACGCTGTGAATGG | 147 | A/G | NM_001129992  (3.42E-33) | Otolith matrix macromolecule |
|  |  |  | R: TGGCAAGAGAGGGTTAAGGA |  |  |  |
| *One110036* | 110036 | 479 | F: GGAGACCAGGAGGGAGATGT | 136 | A/G | EF055889  (0) | Mitochondrion, complete genome |
|  |  |  | R: AGCGACTGATCCCCTATCAA |  |  |  |
| *One72541* | 72541 | 122 | F: GAAAAGGGGCAGAAGTGGAC | 143 | C/G | NM_001141209  (0) | Cell death activator |
|  |  |  | R: TTCAGACAGCCAATGAAATCC |  |  |  |
| *One82766* | 82766 | 142 | F: GAGTGCGGAATTTGTTACCG | 144 | A/T | NM_001140984  (0) | RING finger protein |
|  |  |  | R: CGAACATTCCTTCGTGGATT |  |  |  |
| *One81166* | 81166 | 384 | F: CTATAGCCCAGTATTTGGTTAATTCT | 150 | A/G | No hits |  |
|  |  |  | R: GCAACATAAACAGAACATAACTACTCA |  |  |  |
| *One14410** | 14410 | 533 | F: ACAGACATGCGTTGTGCATT | 148 | A/T | NM001160652  (1.19E-161) | FK506-binding protein |
|  |  |  | R: CACACTCATCTGGCCAACC |  |  |  |
| *One73115* | 73115 | 93 | F: AATGGCCTCCCCTTCACCCC | 77 | A/G | NM001140906  (7.48E-116) | Coiled-coil-helix-coiled-coil-helix domain-containing protein |
|  |  |  | R: TCTGGTCCACTGGGTCCTCCT |  |  |  |
| *One51554** | 51554 | 148 | F: GCCGGCCTGCCCAACCAAAT | 86 | A/G | BT075773  (9.64E-150) | Zymogen granule membrane protein |
|  |  |  | R: CCGTTGAAGCGACCGCTGAGG |  |  |  |
| *One47246* | 47246 | 463 | F: AGACTGGTTAACATGACAGTGGAACA | 76 | A/G | NM_001140966  (0) | Dehydrogenase/reductase SDR family |
|  |  |  | R: AGAAAGGGTTGACTGCTGCGT |  |  |  |
| *One73476* | 73476 | 793 | F: GGTACAGTGATGTTGAGCTCATGG | 106 | A/C | BT046613  (0) | Transmembrane protein |
|  |  |  | R: AAGTCACGGAAATGGCTCACG |  |  |  |
| *One23430* | 23430 | 427 | F: CTGCTCGCCACTGGAGTCAT | 68 | C/T | BT045914  (0) | Vesicle-associated membrane protein |
|  |  |  | R: TGAGCTTTTGTGGGTGTGGGA |  |  |  |
| *One19536* | 19536 | 453 | F: GTTTCGCCAGAGGATGCTATCAAATGT | 94 | A/G | NM001124296  (0) | Initiation factor 2 alpha subunit |
|  |  |  | R: ACTCCAGCACCTCAGCCACG |  |  |  |
| *One50031* | 50031 | 215 | F: CACCACCGTGGGGTCTCTCA | 77 | C/G | NM001141230  (9.38E-174) | Ubiquitin-like protein |
|  |  |  | R: CACCTGACAGCCTCTGCCTTTC |  |  |  |
| *One51420* | 51420 | 245 | F: CGGGCTTCTACATCAGATAGCAAAT | 98 | G/T | NM001140831  (0) | Growth arrest and DNA-damage-inducible proteins |
|  |  |  | R: CCGGAGTTTTCTCGCTTTCCC |  |  |  |
| *One113434** | 113434 | 135 | F: TCAACCGTGTGGAGGTCAAGGT | 99 | C/G | NM001165073  (0) | Mortality factor 4-like protein |
|  |  |  | R: GGTGAAAAAGCTGCTTCTGTCTGGT |  |  |  |
| *One74836** | 74836 | 536 | F: CCTGTTCTTAAATCTGCCGCTGCTC | 107 | A/C | NM001124226  (3.50E-153) | B-type natriuretic peptide |
|  |  |  | R: GGCCTATAGCCTTAAAGGGACTCACC |  |  |  |
| *One74160** | 74160 | 513 | F: TCAGTGCTACAAGATAGTGGTCAAC | 75 | A/C | NM001124191  (0) | Galectin like protein |
|  |  |  | R: ACCTGCTGGAGGAGGGTG |  |  |  |
| *One107712** | 107712 | 627 | F: AGGACACTACACACCCGCCA | 86 | C/G | NM001165124  (0) | CF203 protein |
|  |  |  | R: GCTGAATTCCAGCGTTTAGGAAGC |  |  |  |
| *One72024* | 72024 | 616 | F: TCTCCATTCCCTTTCTCATCAGCTACA | 88 | C/T | NM_001140945  (0) | Nuclear gene encoding mitochondrial protein |
|  |  |  | R: ACACCCTTGGGAGTCCCTGTG |  |  |  |
| *One22742** | 22742 | 1142 | F: TCCCCAGTTAAGCTGGAGGTTCAGG | 96 | G/T | BT048797  (0) | Mitotic-specific cyclin-B1 putative mRNA |
|  |  |  | R: GGCCTGGCACAGTTCAATGGGA |  |  |  |
| *One22253** | 22253 | 501 | F: CATGAAGCTGGGCGCTGGGT | 77 | C/T | BT074272  (0) | Mitochondrial precursor |
|  |  |  | R: TGAACTTAGCCGTGTCCAGTCCTACG |  |  |  |
| *One72037** | 72037 | 1869 | F: GAAAACGATGATGACTTCAACTGTGG | 70 | C/T | NM001193497  (0) | selenoprotein |
|  |  |  | R: GTCTGCCACAGCTGCTCC |  |  |  |
| *One74292* | 74292 | 163 | F: GAGAGACCTAAAGCCTGCTCCG | 87 | A/G | NM001124195  (0) | Rhamnose binding lectin |
|  |  |  | R: GGTCGCCACATCACACTTCTCA |  |  |  |
| *One72741** | 72741 | 846 | F: AGCCTGACTCCCCAGGACT | 72 | A/G | BT074218  (0) | Pallidin putative mRNA |
|  |  |  | R: CTCTTCAATAGGGACTGATTTCTCTGC |  |  |  |
| *One24190* | 24190 | 432 | F: CCAAGGGTTCGAGGTGGCCC | 87 | C/T | NM001146585  (0) | Transmembrane protein |
|  |  |  | R: TGGCAGTCCCCATGGTTACGGTT |  |  |  |

*Contig consensus sequence was reversed to create the primers
