# Supplementary material for: Transcriptome-wide comparison of sequence variation in divergent ecotypes of kokanee salmon
Source: BMC Genomics. 2013 May 7;14:308. doi: 10.1186/1471-2164-14-308 (PMC3653777; doi:10.1186/1471-2164-14-308)
Supplement: Additional file 7 — Sample collection. Containing the sampling locations of all kokanee used to generate the two pooled transcriptome libraries. [file 1471-2164-14-308-S7.doc]

**Additional File 6:** Collection information of all individuals used for each pooled cDNA library

| Ecotype | Spawning Site | Location | Sex |
| --- | --- | --- | --- |
|  |  |  |  |
| Stream-Spawner | Mission Creek | 49.878500°  -119.415458° | 1. Female |
|  |  |  | 2. Male |
|  | Penticton Creek | 49.497669°  -119.587157° | 3. Male |
|  |  |  | 4. Female |
|  | Peachland Creek | 49.741499°  -119.762465° | 5. Male |
|  |  |  | 6. Male |
|  | Powers Creek | 49.815830°  -119.623955° | 7. Male |
|  |  |  | 8. Male |
|  |  |  |  |
| Shore-Spawner | Southeast Shore | 49.732846°  -119.718339° | 1. Female |
|  |  |  | 2. Male |
|  |  |  | 3. Female |
|  | Northeast Shore | 50.211450°  -119.393572° | 4. Male |
|  |  |  | 5. Female |
|  | Northwest Shore | 49.996623°  -119.493216° | 6. Male |
|  |  |  | 7. Male |
|  |  |  | 8. Female |
|  |  |  |  |
